# Different Effects of Eicosapentaenoic and Docosahexaenoic Acids on Atherogenic High-Fat Diet-Induced Non-Alcoholic Fatty Liver Disease in Mice

**DOI:** 10.1371/journal.pone.0157580

**Published:** 2016-06-22

**Authors:** Noriko Suzuki-Kemuriyama, Takashi Matsuzaka, Motoko Kuba, Hiroshi Ohno, Song-iee Han, Yoshinori Takeuchi, Masaaki Isaka, Kazuto Kobayashi, Hitoshi Iwasaki, Shigeru Yatoh, Hiroaki Suzuki, Katsuhiro Miyajima, Dai Nakae, Naoya Yahagi, Yoshimi Nakagawa, Hirohito Sone, Nobuhiro Yamada, Hitoshi Shimano

**Affiliations:** 1 Department of Internal Medicine (Endocrinology and Metabolism), Faculty of Medicine, University of Tsukuba, 1-1-1 Tennodai, Tsukuba, Ibaraki 305–8575, Japan; 2 Department of Nutritional Science and Food Safety, Faculty of Applied Bioscience, Tokyo University of Agriculture, 1-1-1 Sakuragaoka, Setagaya-ku, Tokyo 156–8502, Japan; 3 International Institute for Integrative Sleep Medicine (WPI-IIIS), University of Tsukuba, 1-1-1 Tennodai, Tsukuba, Ibaraki 305–8575, Japan; 4 Department of Internal Medicine, Faculty of Medicine, Niigata University, 1–754 Asahimachi, Niigata 951–8510, Japan; 5 AMED-CREST, Japan Agency for Medical Research and Development (AMED), 1-7-1, Ohte-machi, Chiyoda-ku, Tokyo, 100–0004, Japan; INRA, FRANCE

## Abstract

Non-alcoholic fatty liver disease (NAFLD), the hepatic manifestation of metabolic syndrome, can progress to steatohepatitis (NASH) and advanced liver damage, such as that from liver cirrhosis and cancer. Recent studies have shown the benefits of consuming n-3 polyunsaturated fatty acids (PUFAs) for the treatment of NAFLD. In the present study, we investigated and compared the effects of the major n-3 PUFAs—eicosapentaenoic acid (EPA, C20:5) and docosahexaenoic acid (DHA, C22:6)—in preventing atherogenic high-fat (AHF) diet-induced NAFLD. Mice were fed the AHF diet supplemented with or without EPA or DHA for four weeks. Both EPA and DHA reduced the pathological features of AHF diet-induced NASH pathologies such as hepatic lobular inflammation and elevated serum transaminase activity. Intriguingly, EPA had a greater hepatic triacylglycerol (TG)-reducing effect than DHA. In contrast, DHA had a greater suppressive effect than EPA on AHF diet-induced hepatic inflammation and ROS generation, but no difference in fibrosis. Both EPA and DHA could be effective for treatment of NAFLD and NASH. Meanwhile, the two major n-3 polyunsaturated fatty acids might differ in a relative contribution to pathological intermediate steps towards liver fibrosis.

## Introduction

Non-alcoholic fatty liver disease (NAFLD) is the most common chronic liver disease in all contemporary societies [1–4]. NAFLD is characterized by not only steatosis but also lobular inflammation and fibrosis of the liver. Non-alcoholic steatohepatitis (NASH) belongs to the spectrum of NAFLD, develops inflammatory changes, and can sometimes progresses to cirrhosis and hepatocellular carcinoma [5,6]. It has been proposed that NAFLD/NASH is associated with metabolic abnormalities, including obesity, insulin resistance, and dyslipidemia, as well as with cardiovascular disease events [7–9]. The mechanisms of disease progression in NASH are not completely understood yet.

It has been hypothesized that the development of NASH requires two “hits” [10]. In this hypothesis, the first hit represents the development of hepatic steatosis. The second hit involves oxidative stress and proinflammatory cytokines which induce further liver injury. It is also proposed that inflammation results in a stress response of hepatocytes, may lead to lipid accumulation, and thus could precede steatosis in NASH [11–13]. Recently, growing evidence suggests that simple steatosis and NASH are two separate diseases. In this “multiple parallel hit” hypothesis, the accumulated lipotoxic/pro-inflammatory lipid species interact with pro-inflammatory factors to cause progression to NASH, whereas in other cases, the liver develops steatosis and remains free of inflammation [14–16]. Very diverse processes, including toxic lipids, nutrients, and other macrophage- and adipose-derived signals, may represent such inflammatory insults. Thus, a mechanism combining metabolic changes and inflammation is important in early stages of the NASH pathological process.

Recent studies suggest that types of fat sensitize hepatocytes to the inflammation progress [17]. We recently reported that a change in long-chain fatty acid composition via Elovl6 modulates the progress of NASH [18,19]. Dietary fish oil, which contains large amounts of polyunsaturated fatty acids (PUFAs) of the n-3 family, improves whole-body lipid metabolism, inflammation, and cardiovascular disease [20–23]. In addition, we and others have reported that fish oil suppresses hepatosteatosis [24–26], although there are compelling reports for human studies against the effects of these PUFAs on hepatosteatosis or fibrosis [27,28].

The major long chain n-3 PUFAs are eicosapentaenoic acid (EPA, C20:5 n-3) and docosahexaenoic acid (DHA, C22:6 n-3), derived principally from fish oil. Although several reports have described the preventive effects of EPA or DHA in experimentally induced NAFLD [27–31], most studies have used either EPA or DHA, and few studies have compared the efficacy of EPA and DHA in improving NAFLD. Recently, Depner *et al*. reported that the capacity of dietary DHA to suppress hepatic inflammation, fibrosis, and oxidative stress was significantly greater than that of dietary EPA in a low-density lipoprotein receptor (LDLR) knockout mouse model of western diet-induced NASH [32]. Moreover, Watanabe *et al*. reported that EPA supplementation reduced hepatic cholesterol ester accumulation but aggravated liver injury and associated inflammatory responses in a mouse model of sodium cholate-supplemented high-fat diet-induced steatohepatitis [33]. In the present study, we investigated and compared the effects of the major n-3 PUFAs, EPA and DHA, on attenuation of atherogenic high-fat (AHF) diet-induced NAFLD.

## Materials and Methods

### Materials

EPA ethyl ester and DHA ethyl ester (95% grade) were provided by Mochida Pharmaceutical (Tokyo, Japan). Standard laboratory chow (composed of 60% carbohydrate, 13% fat, and 27% protein on a caloric basis) were purchased from Oriental Yeast (Tokyo, Japan). An AHF diet was prepared by modification of a high-fat–high-sucrose diet with 1.25% cholesterol and 0.5% cholate (Oriental Yeast, Tokyo, Japan) [18]. The dietary component of AHF and fatty acid composition of each diet are presented in S1 and S2 Tables, respectively.

### Animals

All animal husbandry and experiments were performed in compliance with the guiding principle of the University of Tsukuba and were approved by the Animal Experiment Committee of the University of Tsukuba. Thirteen-week-old male C57BL/6J mice were purchased from Clea Japan and adapted to the environment for one week prior to study. Mice were housed in colony cages with a 12-h light/12-h dark cycle and given free access to food and water. At 14 weeks of age, mice were randomly allocated to the following four diet groups of 12–15 animals each. The groups were assigned to the standard laboratory rodent chow, the AHF diet, the AHF diet supplemented with 5% EPA, or the AHF diet supplemented with 5% DHA. The diets were changed every other day to prevent the formation of oxidation products. Mice were given each diet for four weeks and body weight was monitored weekly. At the end of the period, all mice were sacrificed in the early light phase in a non-fasting state.

### Histological analysis

Livers were removed, fixed in 10% buffered formalin, embedded in paraffin, and cut into 4-μm-thick sections for haematoxylin and eosin (H&E) staining. Immunohistochemical staining for F4/80 (Abcam, Cambridge, UK) and Nε-(Hexanoyl) Lysine (HEL, JAICA, Shizuoka, Japan) were also performed. Briefly, deparaffinized sections were heated in citrate buffer to accomplish antigen retrieval. Endogenous peroxidase activity was blocked with 1% hydrogen peroxide in PBS for 30 min. Next, the sections were incubated with a primary antibody overnight at 4°C, followed by treatment with a labeled polymer anti-rat IgG or anti-mouse IgG (Nichirei Bioscience, Tokyo, Japan) for 30 min at room temperature. The antibody-peroxidase complex was visualized using diaminobenzidine (Nichirei Bioscience, Tokyo, Japan), and sections were counterstained with hematoxylin. Sirius red staining was performed as described previously [18].

### Blood chemistries and liver lipid analyses

Blood samples were obtained by orbital bleeding. Enzymatic assays for glucose, triglycerides (TG), total cholesterol (T-Cho), free fatty acids (FFA), alanine aminotransferase (ALT), and aspartate aminotransferase (AST) levels were performed using colorimetry test kits purchased from Wako Pure Chemical Industries (Osaka, Japan). Plasma insulin levels were determined using a mouse insulin ELISA kit (Shibayagi, Gunma, Japan) according to the manufacturer’s protocol. Hepatic TG and T-Cho levels were measured as described previously [34].

### Fatty acid composition of liver

Total lipids in liver were extracted by Bligh-Dyer’s procedure, and the relative abundance of each FA was quantitatively measured by LC–MS/MS as previously reported (JCL Bioassay Laboratories, Osaka, Japan) [35]. Briefly, internal solution was spiked to all samples (20 μl), obtained by lipid extraction as described above, and to calibration standard samples, and the samples were evaporated by nitrogen gas. Following the addition of acetonitrile/6N HCl (90/10, v/v), samples were incubated at 100°C for 45 min. Finally, liquid-liquid extraction with ethyl acetate was performed and the reconstituted samples were injected into an optimized LC–MS/MS system. LC was performed using an ACQUITY UPLC with a YMC-Triart C18 (YMC Co., Ltd., Kyoto, Japan) and a linear gradient of a combination of two mobile phases, and an API4000 triple quadrupole tandem mass spectrometer was used as a detector using atmospheric pressure chemical ionization in negative ionization with selected reaction monitoring mode. The relative contents of the respective FAs were calculated from the LC–MS/MS chromatogram.

### RNA extraction and quantitative real-time PCR

Total RNA was extracted from livers using Sepasol reagent (Nacalai Tesque, Kyoto, Japan) and reverse-transcribed using the PrimeScript RT Master kit (Takara Bio Inc., Shiga, Japan) according to the manufacturer’s protocols. Quantitative real-time PCR (qPCR) was performed using SYBR Premix Ex Taq (Takara Bio Inc. Shiga, Japan) and specific primer sets with the Thermal Cycler Dice Real Time System Single (Takara Bio Inc. Shiga, Japan). Primer sequences for qPCR in this study have been described previously [18,19,34,36]. The expression levels of mRNA were normalized to those of cyclophilin mRNA.

### Immunoblotting

Immunoblotting was performed as described previously [34]. Aliquots of nuclear extract (25 μg) and total lysate (50 μg) proteins extracted from livers were loaded onto 10% SDS-PAGE gels and transferred to PDVF membranes (Millipore, Darmstadt, Germany). The membranes were probed with anti-SREBP-1 (Santa Cruz Biotechnology, Dallas, USA), lamin A/C, phospho-JNK, and total JNK (Cell Signaling Technology, Denvers, USA) followed by horseradish peroxidase (HRP)-conjugated anti-mouse or rabbit IgG (Cell Signaling Technology, Denvers, USA). Immune complexes were visualized using enhanced chemiluminescence (GE Healthcare Japan, Tokyo, Japan).

### Determination of hepatic 8-OHdG levels in liver

DNA was isolated from the livers using the DNA Extractor TIS Kit (Wako, Osaka, Japan), and hepatic 8-OHdG levels were determined as described using a competitive ELISA kit (8-OHdG Check; Japan Institute for the Control of Aging, Fukuroi, Japan).

### Statistical analysis

Values are expressed as means ± SEM. Analysis of variance (ANOVA) followed by Tukey-Kramer test was used to assess differences among groups. Differences were considered significant at p < 0.05.

## Results

### Effects of EPA and DHA on hepatic lipid accumulation and liver injury in AHF diet-fed mice

The AHF diet is a useful dietary model for NASH with progression from NAFLD [18,37]. To investigate the effect of EPA and DHA on AHF diet-induced NAFLD, C57BL6/J mice were fed a normal chow, AHF diet, or AHF diet supplemented with EPA or DHA for four weeks. Table 1 compares body weight, tissue weight, and plasma metabolic parameters in C57BL/6J mice fed these diets. The mice in the four dietary groups showed similar body weights. Feeding C57BL/6J mice the AHF diet for four weeks slightly increased the liver/body weight ratio compared with the chow-fed C57BL/6J mice. This ratio was significantly increased in AHF + EPA group compared to the other groups. The AHF diet elevated total cholesterol levels and decreased plasma TG levels. Hypercholesterolemia was attenuated in both AHF + EPA and AHF + DHA groups. The plasma FFA level of the AHF + EPA group was the lowest among the four groups. There was no significant intergroup difference in plasma glucose and insulin levels.

**Table 1:** Phenotypic comparison of C57BL/6J mice fed the chow, AHF, AHF + EPA, and AHF + DHA diet for 4 weeks.

Parameters	Chow	AHF	AHF+EPA	AHF+DHA
Body weight (g)	27.5 ± 0.52	25.3 ± 0.57	25.9 ± 0.51	26.2 ± 0.40
Liver weight (%body weight)	4.77 ± 0.11	5.14 ± 0.12	5.39 ± 0.21*	4.97 ± 0.07
eWAT weight (%body weight)	1.41 ± 0.08	1.09 ± 0.09	1.15 ± 0.08 ^##^	1.38 ± 0.06
Glucose (mg/dl)	164 ± 9.4	169 ±9.0	152 ± 6.7	149 ± 5.8
Insulin (ng/ml)	0.72 ± 0.21	0.31 ± 0.07	0.52 ± 0.18	0.61 ± 0.23
Plasma T-Cho (mg/dl)	79.3 ± 2.9	143.5 ± 7.0 **	114.1 ± 5.5 **^,^ ^##^	124.7 ± 5.4 **^,^ ^##^
Plasma TG (mg/dl)	85.7 ± 5.8	54.6 ± 5.3 **	56.0 ± 4.5 **	64.0 ± 5.1 **
Plasma FFA (mM)	0.55 ± 0.06	0.48 ± 0.03	0.38 ± 0.03 *	0.45 ± 0.02

Values are mean ± SEM. n = 12–15 per group. eWAT, epididymal white adipose tissue; T-Cho, total cholesterol; TG, triglyceride; FFA, free fatty acid; AHF + EPA, atherogenic high-fat diet supplemented with EPA; AHF + DHA, atherogenic high-fat diet supplemented with DHA.

* *P* < 0.05 vs chow group

** *P* < 0.01 vs chow group

## *P* < 0.01 vs AHF group.

**Fig 1:**
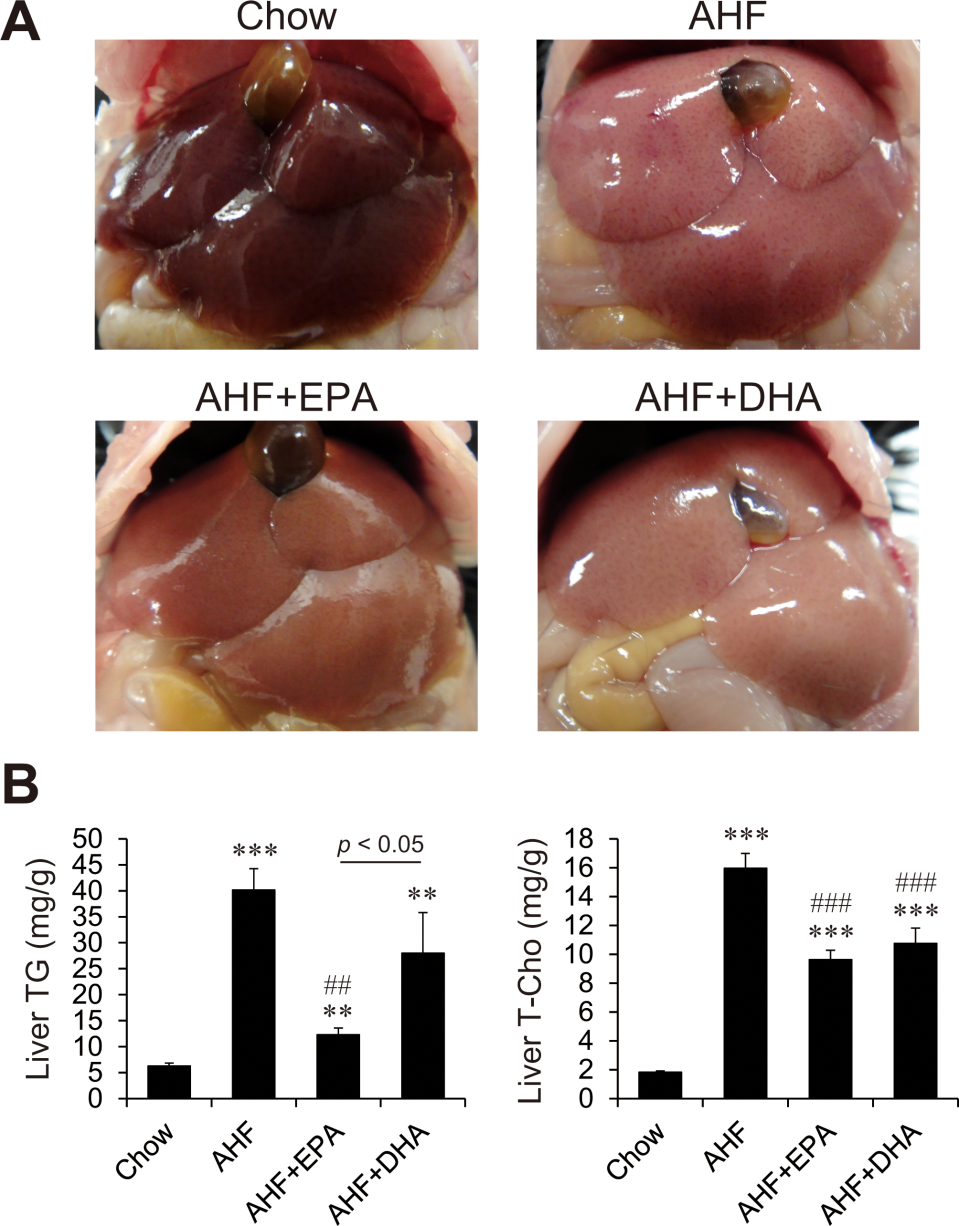
Effect of EPA and DHA on hepatic lipid content in mice fed AHF diet. Liver macroscopic picture (A) and hepatic triglyceride (TG) and total cholesterol (T-Cho) levels (B) in mice fed a normal chow, an AHF diet, or an AHF diet supplemented with EPA or DHA for four weeks. n = 12–15 per group. ** p < 0.01, *** p < 0.001 versus chow group; ## p < 0.01, ### p < 0.001 versus AHF group.

Images of the opened abdominal cavities of mice showed that, unlike that of the chow diet group, the liver of the AHF diet group had a white tinge and was enlarged (Fig 1A). However, these changes in hepatic abnormalities induced by the AHF diet were efficiently attenuated by the addition of EPA or DHA to the AHF diet, as reflected in changes in liver appearance. Hepatic triacylglycerol (TG) levels in the AHF group were markedly elevated (Fig 1B). The effects of EPA or DHA on liver TG accumulation differed. EPA prevented AHF diet-induced increase in liver TG, but DHA did not significantly prevent the AHF diet-induced increase. Hepatic total cholesterol (T-Cho) levels were also markedly increased in the AHF group and hepatic cholesterol accumulation was equally decreased in both AHF + EPA and AHF + DHA groups (Fig 1B).

### EPA and DHA prevent AHF diet-induced hepatic inflammatory features

**Fig 2:**
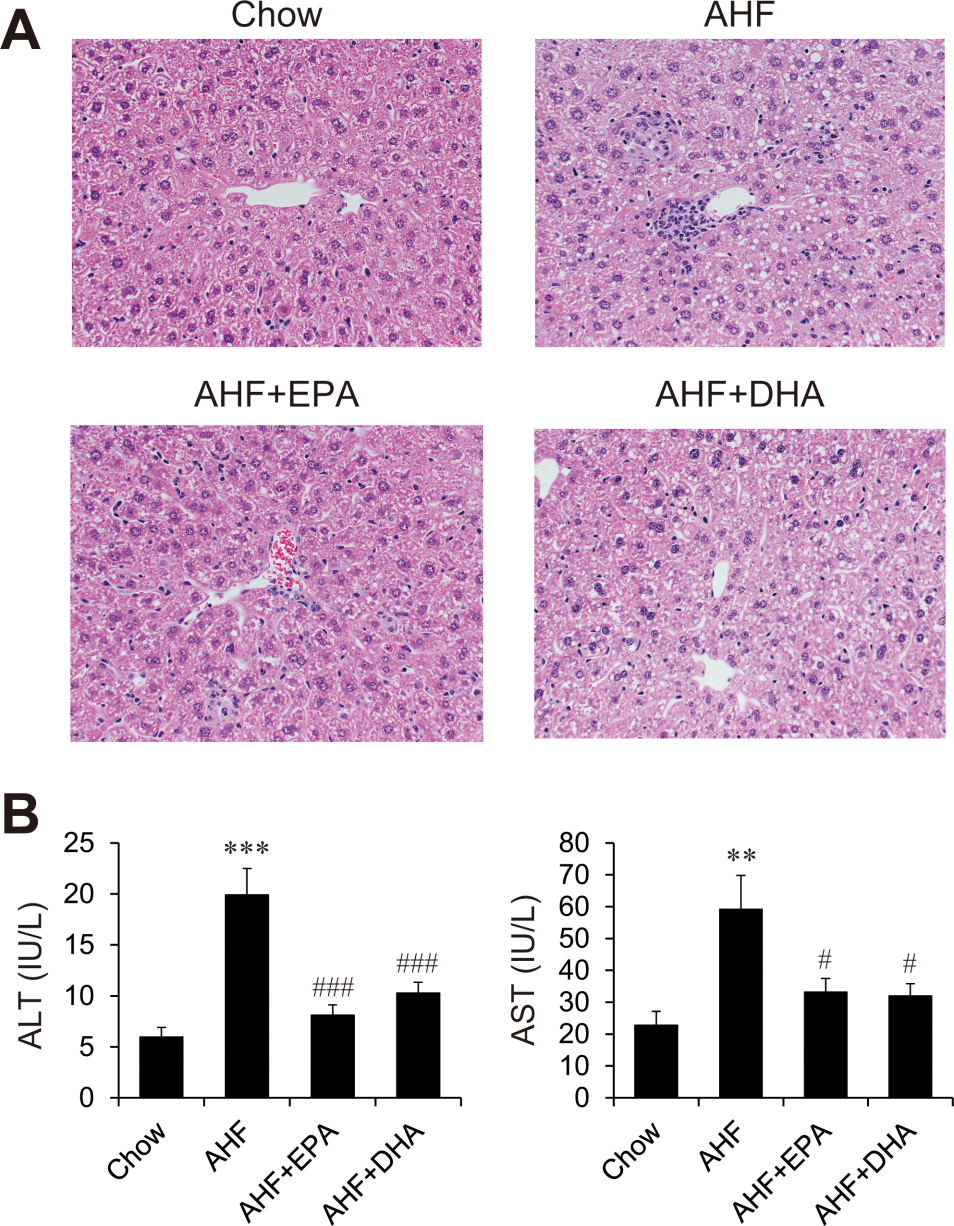
Attenuated hepatic inflammation and liver injury in mice fed AHF supplemented with EPA or DHA. Hematoxylin and eosin (H&E) staining of liver sections from representative mice from each treatment group (A), and plasma alanine aminotransferase (ALT) and aspartate aminotransferase (AST) levels (B). n = 11–15 per group. ** p < 0.01, *** p < 0.001 versus chow group; # p < 0.05, ### p < 0.001 versus AHF group.

The histology of livers from the four groups is shown in Fig 2A. Inflammatory cell infiltration including macrophage and lymphocyte were observed in centrilobular area of the AHF compared with the chow diet group. The hepatic inflammation induced by the AHF diet was efficiently attenuated by the addition of EPA or DHA to the AHF diet, as reflected in significantly fewer hepatic lobular inflammatory clusters than in the AHF diet group. Slight to moderate centrilobular fatty change including microvesicular or large droplet were observed in the AHF or DHA compared the chow diet group, and very slight fatty change was observed in the EPA. In addition, EPA or DHA administration was found to improve liver injury as assessed by serum transaminase activities (Fig 2B). Feeding mice with the AHF diet resulted in approximately 4- and 3-fold increases in levels of plasma ALT and AST, respectively. These biochemical liver injury markers induced by AHF diet were similarly reduced by dietary EPA and DHA.

### Effects of AHF diet supplemented with EPA or DHA on hepatic fatty acid composition

**Fig 3:**
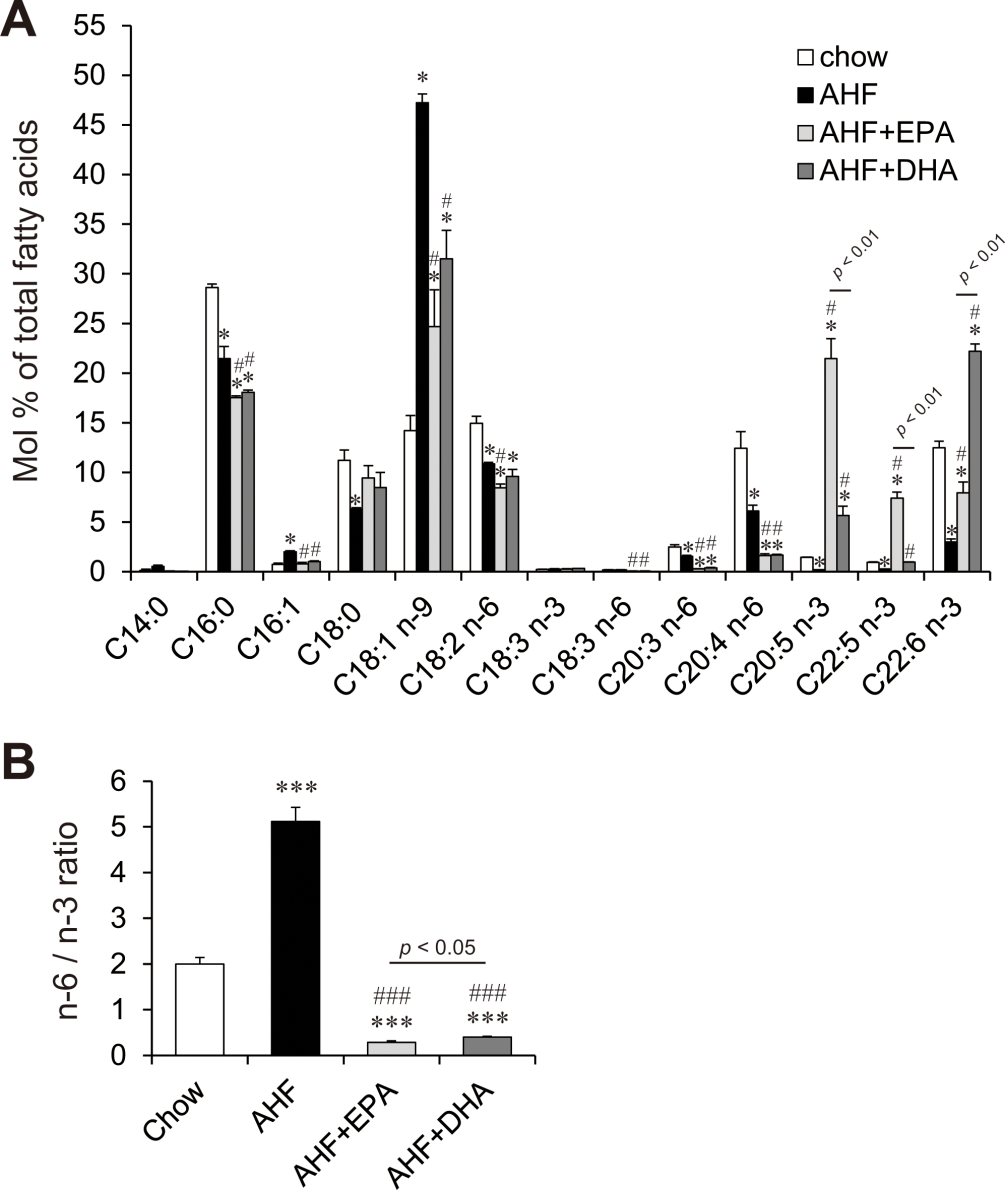
Hepatic fatty acid composition in mice fed normal chow, AHF, AHF + EPA and AHF + DHA diets for four weeks. Hepatic fatty acid composition (A) and the n-6/n-3 fatty acid ratio (B) in livers of mice fed a normal chow, an AHF diet, or an AHF diet supplemented with EPA or DHA for four weeks. n = 3–4 per group. * p < 0.05, *** p < 0.001 versus chow group; # p < 0.05, ### p < 0.001 versus AHF group.

In hepatic FA composition analysis, the livers of the AHF group showed increased amounts of palmitoleic acid (C16:1 n-7) and oleic acid (C18:1 n-9) but significantly reduced amounts of palmitic acid (C16:0), stearic acid (C18:0), linoleic acid (C18:2 n-6), dihomo-gamma-linolenic acid (C20:3 n-6), arachidonic acid (C20:4 n-6), EPA (C20:5 n-3), docosapentaenoic acid (C22:5 n-3), and DHA (C22:6 n-3) in comparison with the chow group (Fig 3A). The amount of hepatic EPA of AHF + EPA group was increased 3.9- and 29-fold in comparison with the chow and AHF groups, respectively. Dietary DHA also clearly increased hepatic DHA content by 1.8- and 7.4-fold compared with the control and AHF groups, respectively. The addition of EPA or DHA to the AHF diet similarly reduced hepatic palmitic acid, palmitoleic acid, and oleic acid. The hepatic levels of the n-6 series of PUFA [arachidonic acid (C20:4 n-6) and its precursor, dihomo-gamma-linolenic acid (C20:3 n-6)] were strongly decreased in both AHF + EPA and AHF + DHA groups in comparison with the AHF group. Mice fed AHF + DHA showed increased hepatic EPA, and mice fed AHF + EPA showed increased hepatic docosapentaenoic acid (DPA) and DHA content compared with the AHF group to the similar extent. The proportion of hepatic n-6/n-3 FA was significantly increased in AHF group as compared with chow group (Fig 3B). The hepatic n-6/n-3 FA ratio was strongly decreased in the AHF + EPA and AHF + DHA groups in comparison with the AHF group. This ratio for the AHF + EPA group was slightly but significantly lower than that of AHF + DHA group.

### Effects of AHF diet supplemented with EPA or DHA on hepatic mRNA and protein levels involved in fatty acid and cholesterol metabolism

To explore the molecular mechanisms underlying the different effects of the EPA and DHA on AHF diet-induced NAFLD, mRNA and protein levels of candidate genes in livers from the chow, AHF, AHF + EPA, and AHF + DHA groups were measured by quantitative real-time polymerase chain reaction (qPCR) and Western blotting.

**Fig 4:**
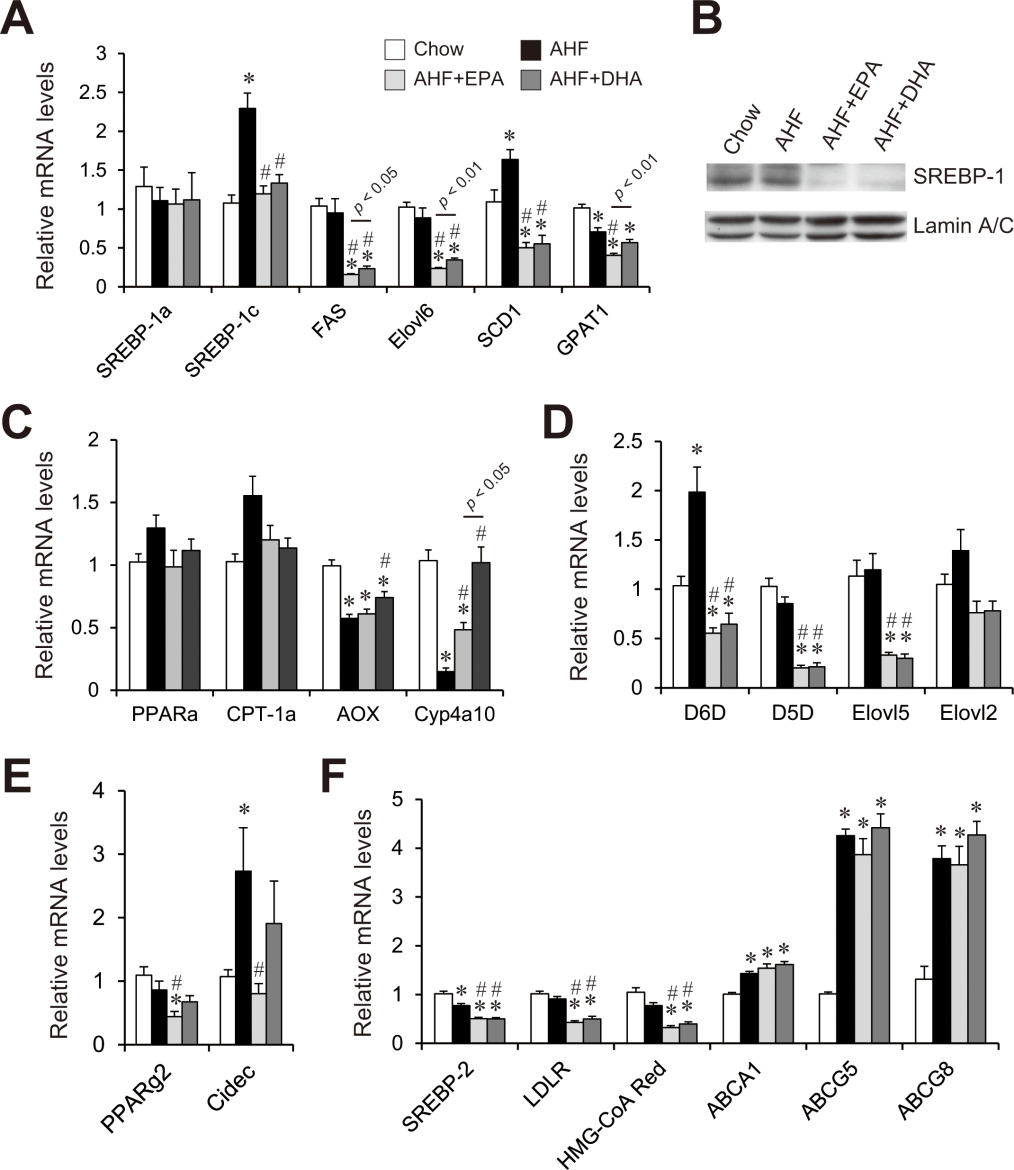
Effect of AHF diet supplemented with EPA or DHA on hepatic lipid metabolism-related mRNA and protein levels. For gene expression and immunoblot analyses, livers were collected from the mice fed a normal chow, an AHF diet, or an AHF diet supplemented with EPA or DHA for four weeks. Quantitative real-time PCR of genes involved in fatty acid and TG synthesis (A), fatty acid oxidation (C), PUFA synthesis (D), lipid storage (E), cholesterol and lipoprotein metabolism (F). Immunoblot analysis for mature SREBP-1 levels (B). n = 13–15 per group. * p < 0.05 versus chow group; # p < 0.05 versus AHF group.

Gene expressions relevant to hepatic synthesis of FAs and TGs (lipogenesis) were measured. Fatty acid synthase (FAS), ELOVL family member 6 (Elovl6), and stearoyl-CoA desaturase-1 (SCD1) play major roles in *de novo* synthesis of FA. In FA synthesis, SREBP-1c and its target SCD-1 mRNA expression levels were significantly increased in the AHF group compared to the chow group (Fig 4A). In contrast, FAS and Elovl6 mRNA levels did not differ in the AHF group compared to the chow group. Addition of EPA or DHA to the AHF diet markedly reduced the levels of SREBP-1c mRNA expression (Fig 4A), mature SREBP-1 protein (Fig 4B), and FA synthesis gene expression in comparison with both chow and AHF diet groups. In TG synthesis, mRNA expression of glycerol-3-phosphate acyltransferase (GPAT)-1 in the AHF group were lower than that in the chow group (Fig 4A). Compared to the AHF + DHA group, the AHF + EPA group showed significantly decreased FAS, Elovl6, and GPAT-1 expression, suggesting that EPA was more effective than DHA in suppressing hepatic FAs and TGs synthesis.

Impairment of FA oxidation contributes to hepatosteatosis. We measured the mRNA abundance of peroxisome proliferator-activated receptor-alpha (PPARα) and its target genes, including carnitine palmitoyltransferase (CPT)-1a, acyl-CoA oxidase (AOX), and cytochrome P450, family 4, subfamily a, polypeptide 10 (Cyp4a10) (Fig 4C). PPARα and CPT-1a mRNA expression levels were not significantly increased in the AHF group compared to the chow group (Fig 4C). n-3 PUFA supplementation is thought to trigger PPARα activation [38,39]. However, the addition of EPA or DHA to the AHF diet did not change the expression of PPARα and CPT-1a. Meanwhile, the expression levels of PPARα-target genes involved in peroxisomal and microsomal FA oxidation such as AOX and Cyp4a10 were significantly decreased in the AHF group compared to the chow group, but the addition of EPA or DHA to the AHF diet recovered the expression of these genes.

Enzymes involved in PUFA synthesis include delta-6 desaturase (D6D), delta-5 desaturase (D5D), Elovl2, and Elovl5. These enzymes are responsible for the conversion of C18:2 n-6 and C18:3 n-3 to C20–22 n-6 and n-3 PUFAs, respectively [40,41]. The hepatic expression of D6D was significantly increased in the AHF group compared to the chow group (Fig 4D). In contrast, D5D, Elovl5, and Elovl2 mRNA levels did not differ in the AHF group compared to the chow group. The mRNA levels of these genes were similarly suppressed in livers of the AHF + EPA and AHF + DHA groups compared to those of the chow and AHF groups.

In lipid storage, cell-death-inducing DFFA-like effector C (Cidec) mRNA expression levels were significantly increased in the AHF group compared to the chow group (Fig 4E). Compared to the AHF group, the AHF + EPA group showed significantly decreased PPARγ2 and Cidec expression, whereas the AHF + DHA group did not suppress the expression of these genes, suggesting that EPA was more effective than DHA in suppressing hepatic lipid storage.

Because hepatic cholesterol content was significantly increased in AHF diet-fed mice and was moderately decreased in the AHF + EPA and AHF + DHA groups (Fig 1B), we investigated the pathways controlling hepatic cholesterol content (Fig 4F). In cholesterol synthesis and uptake, the mRNA abundances of SREBP-2 and its target, low-density lipoprotein receptor (LDLR) and 3-hydroxy-3-methylglutaryl-CoA (HMG-CoA Red) were significantly decreased in both AHF + EPA and AHF + DHA groups compared with the chow and AHF diet groups. Of the lipoprotein metabolism examined, the expression of ATP-binding cassette (ABC) transporters A1 (ABCA1), ABCG5, and ABCG8 were increased in AHF, AHF + EPA, and AHF + DHA diet groups compared with the control group (Fig 4F). However, no difference between EPA and DHA administration was observed.

### Effects of AHF diet supplemented with EPA or DHA on the development of hepatic inflammation

**Fig 5:**
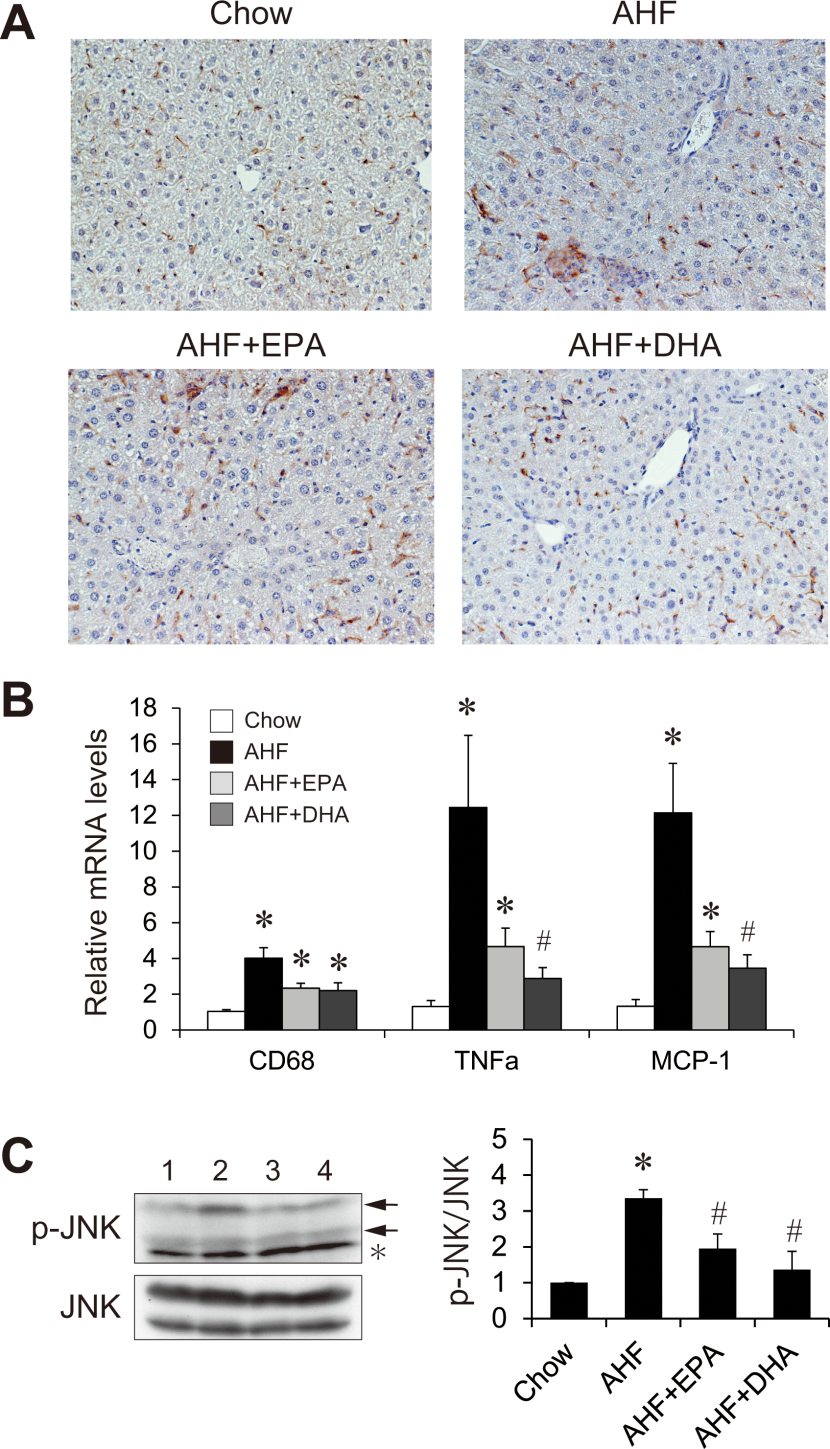
Effect of AHF diet supplemented with EPA or DHA on hepaticinflammation. Representative immunohistochemical staining for F4/80 in liver section form mice fed a normal chow, an AHF diet, or an AHF diet supplemented with EPA or DHA for four weeks (A). Quantitative real-time PCR of genes involved in inflammation in the livers of mice fed a normal chow, an AHF diet, or an AHF diet supplemented with EPA or DHA for four weeks (B). Immunoblot analysis of phosphorylated JNK and total JNK levels and the ratio between phosphorylated and total JNK by densitometry analysis (C). n = 13–15 per group. * p < 0.05 versus chow group; # p < 0.05 versus AHF group.

Hepatic damage involves increased hepatic inflammation and cell death. We analyzed the effects of EPA or DHA supplementation on the AHD diet-induced hepatic inflammation (Fig 5A). Immunohistochemical staining of liver sections for F4/80, a marker for Kupffer cells, revealed that Kupffer cell accumulation and hypertrophied macrophages were markedly greater in the AHF group compared to the chow group, whereas these cells were decreased in livers of the AHF + EPA and AHF + DHA groups compared to those of the AHF groups. In agreement with this observation, the expressions of CD68, tumor necrosis factor alpha (TNFα) and monocyte chemoattractant protein 1 (MCP-1), early markers of inflammation, were strongly upregulated after AHF diet feeding (Fig 5B). The AHF + DHA but not the AHF + EPA diet significantly attenuated the induction of TNFα and MCP-1 expression relative to the AHF group. To test whether the changes observed above for mRNA translated into changes of the corresponding proteins, we performed western blotting of liver lysates (Fig 5C). AHF diet-fed mice showed significantly increased c-Jun N-terminal kinase (JNK) phosphorylation than chow-fed mice. The AHF + EPA and AHF + DHA groups showed significantly decreased the AHF diet-induced JNK activation. These observations suggest that EPA and DHA ameliorate AHF diet-induced hepatic inflammation, which was slightly prominent with DHA.

### Effects of AHF diet supplemented with EPA or DHA on the hepatic oxidative stress

**Fig 6:**
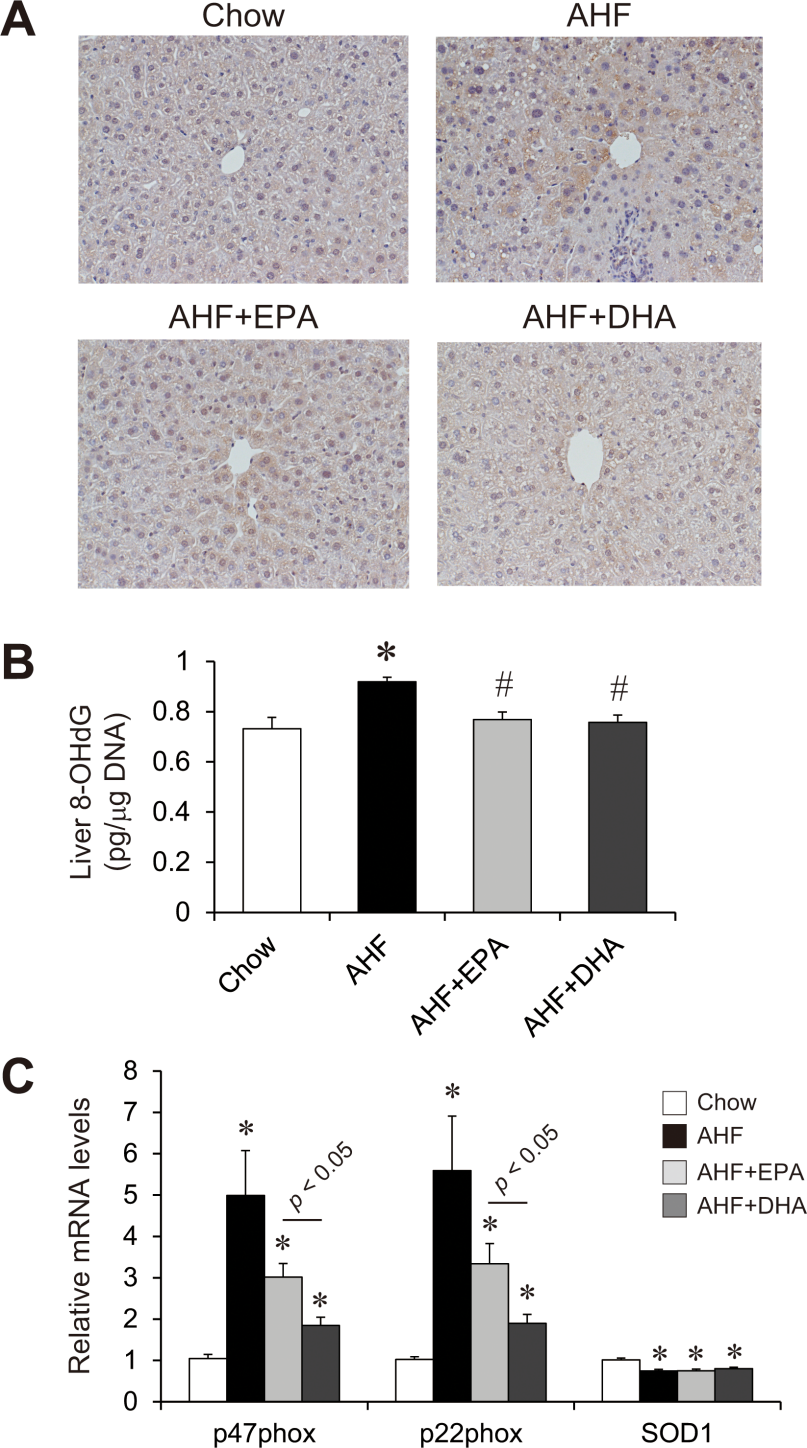
Effect of AHF diet supplemented with EPA or DHA on hepatic oxidative stress. Representative immunohistochemical staining for Nε-(Hexanoyl) Lysine in liver section form mice fed a normal chow, an AHF diet, or an AHF diet supplemented with EPA or DHA for four weeks (A). Liver 8-OHdG levels in mice fed a normal chow, an AHF diet, or an AHF diet supplemented with EPA or DHA for four weeks (B). Quantitative real-time PCR of genes involved in oxidative stress in the livers of mice fed a normal chow, an AHF diet, or an AHF diet supplemented with EPA or DHA for four weeks (B). n = 13–15 per group. * p < 0.05 versus chow group; # p < 0.05 versus AHF group.

We next analyzed the effects of EPA or DHA supplementation on the AHD diet-induced hepatic oxidative stress (Fig 6A). Immunohistochemical staining of liver sections for hexanoyl-lysine (HEL), a marker for early stage of lipid oxidation, revealed that intensity of the HEL-positive hepatocytes was enhanced in the AHF group compared the chow diet group. The AHF + EPA and AHF + DHA groups showed decreased the intensity of the staining, with the effect on DHA group stronger. Consistent with histology stained with HEL-positive cells, hepatic 8-hydroxy-2’-deoxyguanosine (8-OHdG) levels were significantly reduced in the AHF + EPA and AHF + DHA groups compared with the AHF group (Fig 6B). Moreover, the expression of p47phox and p22phox, genes associated with reactive oxygen species (ROS) production, was also dramatically up-regulated in the AHF group (Fig 6C). The induction of p47phox and p22phox was attenuated in the AHF + DHA group but not in the AHF + EPA group. The expression of superoxide dismutase 1 (SOD1), a gene associated with reactive oxygen species (ROS) elimination, was slightly decreased in the AHF group, and addition of EPA or DHA to the AHF diet did not change the expression of SOD1. These observations suggest that EPA and DHA ameliorate AHF diet-induced hepatic oxidative stress, which was more prominent with DHA.

### Effects of AHF diet supplemented with EPA or DHA on the AHF diet-induced hepatic fibrogenesis

**Fig 7:**
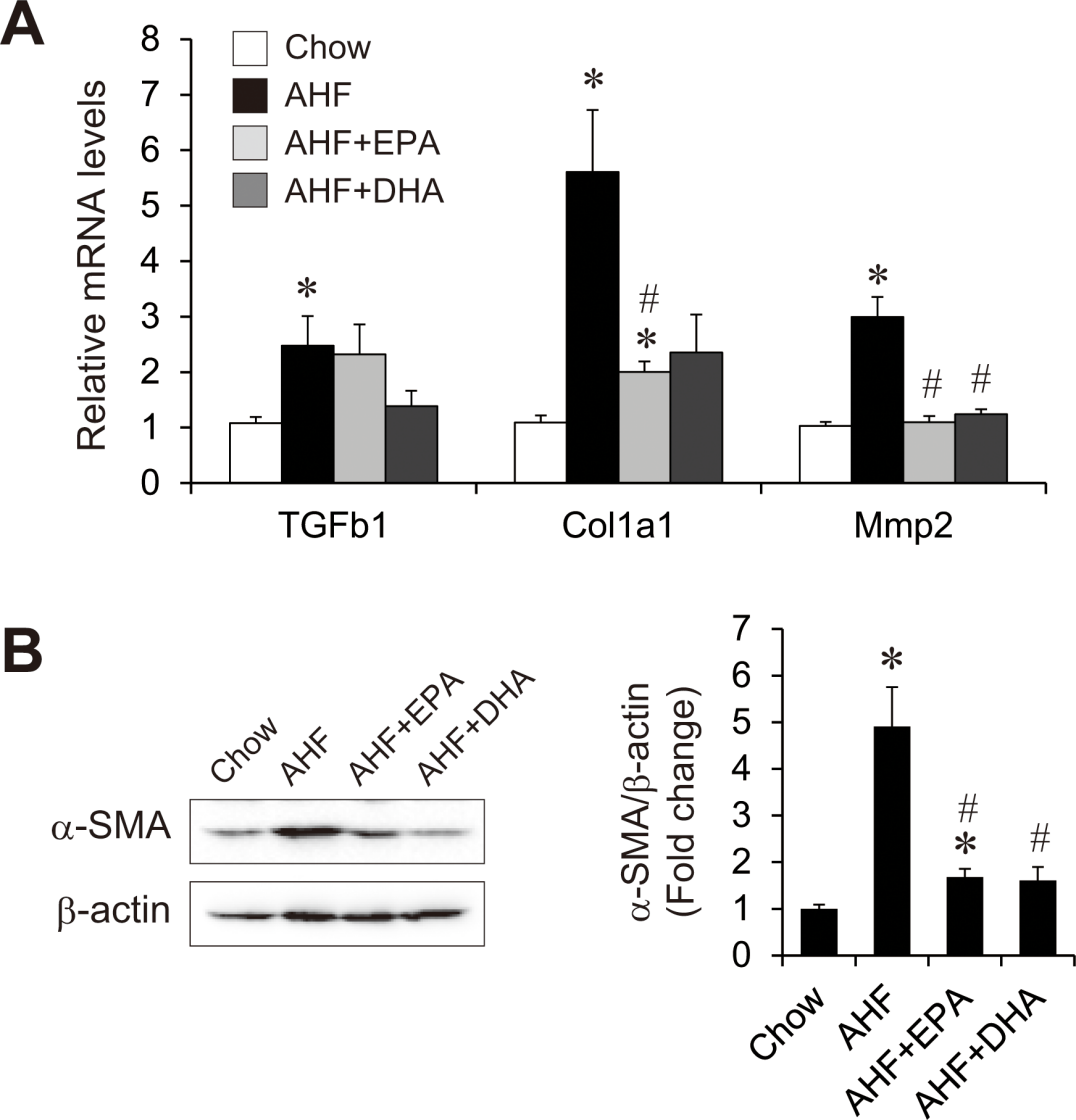
Effect of AHF diet supplemented with EPA or DHA on hepatic fibrogenesis. Quantitative real-time PCR of genes involved in fibrogenesis in the livers of mice fed a normal chow, an AHF diet, or an AHF diet supplemented with EPA or DHA for four weeks (A). Immunoblot analysis of α-SMA levels and the ratio between α-SMA and β-actin by densitometry analysis (B). n = 13–15 per group. * p < 0.05 versus chow group; # p < 0.05 versus AHF group.

We further investigated the expression profile of hepatic fibrogenic factors that may be implicated in the pathophysiology of NASH. The expression levels of genes associated with fibrosis, such as transforming growth factor beta 1 (TGFβ1), collagen type1a (Col1a1), and matrix metallopeptidase 2 (Mmp2) were markedly increased in the AHF group (Fig 7A). Compared with the AHF group, AHF + EPA group showed significantly decreased Col1a1 and Mmp2 expression but unchanged TGFβ1 expression. The AHF + DHA group showed significantly decreased Mmp2 expression with a tendency to decreased expression of both Col1a1 and TGFβ1 compared with the AHF group. Decreased fibrogenesis by EPA or DHA supplementation was further examined by measuring alpha-smooth muscle actin (α-SMA) levels by western blotting (Fig 7B). AHF diet-fed mice showed significantly increased hepatic α-SMA protein levels than chow-fed mice. The AHF + EPA and AHF + DHA groups showed significantly decreased α-SMA protein levels compared with the AHF group to a similar extent after loading correction. Fibrosis was also confirmed by Sirius Red staining (S1 Fig). Sirius Red positive fibrosis was slightly observed in centrilobular area and inflammatory foci in the AHF group compared the chow diet group. Tend to decrease of the fibrosis were noted in both EPA and DHA groups compared the AHF group. These results indicate that despite of difference in inflammation and ROS production, both EPA and DHA are similarly effective of suppression of fibrogenic markers and prevention of early fibrosis.

## Discussion

This study aimed to determine whether purified EPA and DHA have different effects on AHF diet-induced NAFLD development in mice. We found that both EPA- and DHA-containing diet significantly reduced AHF diet-induced steatohepatitis. EPA was more effective than DHA for the improvement of AHF diet-induced hepatic TG accumulation. In contrast, DHA was more effective than EPA for the suppression of AHF diet-induced hepatic inflammation and ROS generation.

According to the two-hit theory, hepatic steatosis of any etiology could be the first “hit.” Growing evidence suggests that n-3 PUFAs can regulate hepatocyte lipid accumulation [25,42]. Treatment of mice with n-3 PUFAs suppressed liver steatosis and inhibited hepatic FA synthesis [24,25,43]. In our study, EPA, but not DHA, significantly suppressed AHF diet-induced hepatic TG accumulation. The lipid content of hepatocytes is regulated by the integrated activities of cellular enzymes that catalyze lipid synthesis, uptake, storage, export, and oxidation. As shown in Fig 4, both EPA and DHA equally reduced mature SREBP-1 protein and lipogenic gene expression. However, EPA was more effective than DHA in the reduction of the mRNA expression of FAS, Elovl6, and GPAT1. Recent functional evidence has indicated that Cidec is localized in lipid droplets and plays a key role in the formation of steatosis [44,45]. AHF-induced induction of Cidec expression was significantly suppressed in the AHF + EPA but not the AHF + DHA group. Expression levels of genes involved in β oxidation and PUFA synthesis were similar between the EPA and DHA groups, suggesting that the difference in hepatic TG accumulation between EPA and DHA was not due to the difference in the capacity for hepatic FA oxidation. These findings indicate that the strong hepatic TG-lowering effect of EPA was due to the suppression of lipogenesis and lipid droplet formation. Our findings are consistent with those of a recent study showing that EPA exerts anti-obesity effect in HF/HS-induced obesity mice by suppressing hepatic lipogenesis and steatosis [43].

It has been proposed that hepatic FA composition is an important determinant of NASH progression [18,19,46,47]. EPA and DHA suppressed the AHF diet-induced accumulation of hepatic palmitoleic acid and oleic acid and strongly reduced hepatic arachidonic acid content. AHF or western diet-induced steatohepatitis mice models show markedly elevated hepatic oleic acid content [18,32,37]. Arachidonic acid-derived prostaglandins and related lipid metabolites are active mediators of inflammation [48–50], and EPA and DHA have antagonistic effects on arachidonic acid metabolism [51]. Compared to the chow group, the AHF group showed a significantly increased n-6/n-3 fatty acid ratio. In contrast, both EPA and DHA showed dramatically decreased ratios. These results suggest that both EPA and DHA strongly attenuate AHF diet-induced liver injury by suppressing n-6 PUFA synthesis and metabolism.

Multiple possible sources, such as inflammation and oxidative stress, in the fatty liver may constitute the second “hit” for cellular injury, apoptosis, and fibrosis in NASH [4,15,52]. In our study, hepatic histology and gene expression analysis showed that EPA and DHA attenuated AHF diet-induced hepatic inflammation, oxidative stress, and fibrosis. In no case was DHA less effective than EPA in suppressing hepatic inflammation and oxidative stress. In a recent study, DHA was more effective than EPA in attenuating inflammation, oxidative stress, fibrosis, and hepatic damage in LDLR-deficient mice fed a high-fat, high-cholesterol diet [32]. These results suggested that a reduction in hepatosteatosis is not required for DHA to attenuate AHF diet-induced hepatic damage, such as by inflammation, oxidative stress, and fibrosis. The molecular basis for this difference is currently enigma, it could be related to the molecule features of both lipids that EPA has a higher kinetics of plasma–tissue turnover whereas DHA has trend of retention in tissue [53,54]. In our current experimental setting, dietary administration of EPA or DHA caused considerable increases in hepatic content of the other counterpart in both cases, which might have obscured the potential more discriminative effects, although inter-conversion between EPA and DHA in plasma levels have been under clinical debate. Further studies on longer term are required to conclude the effects of EPA and DHA on liver fibrosis and cancer.

In conclusion, both EPA and DHA improve the pathological features of AHF-induced NASH pathologies. EPA is more effective than DHA in reducing hepatosteatosis. In contrast, DHA is more effective than EPA in attenuating hepatic inflammation and ROS generation. EPA and DHA ameliorate AHF diet-induced NAFLD and NASH pathology towards liver fibrosis by different mechanism in which TG-reducing effect make a large contribution to benefit of EPA and suppressive effect for inflammation or ROS generation make a large contribution to benefit of DHA. Clinical application to humans may select EPA and DHA to fit of the respective pathological conditions. Further studies may shed light on the relationship between the type of lipid accumulated in the liver and the effect of EPA and DHA on NAFLD.

## Supporting Information

S1 Fig Representative Sirius red staining in the liver sections from mice fed a normal chow, an AHF diet, or an AHF diet supplemented with EPA or DHA for four weeks. (PDF)

S1 Table The Composition of the Atherogenic High-Fat (AHF) Diet. (PDF)

S2 Table Fatty acid composition (mol% of total fatty acids) of the chow and AHF diet. (PDF)
