# Supplementary figures and images for: Different Effects of Eicosapentaenoic and Docosahexaenoic Acids on Atherogenic High-Fat Diet-Induced Non-Alcoholic Fatty Liver Disease in Mice
Source: PLoS One. 2016 Jun 22;11(6):e0157580. doi: 10.1371/journal.pone.0157580 (PMC4917109; doi:10.1371/journal.pone.0157580)

# Figure S1

Chow

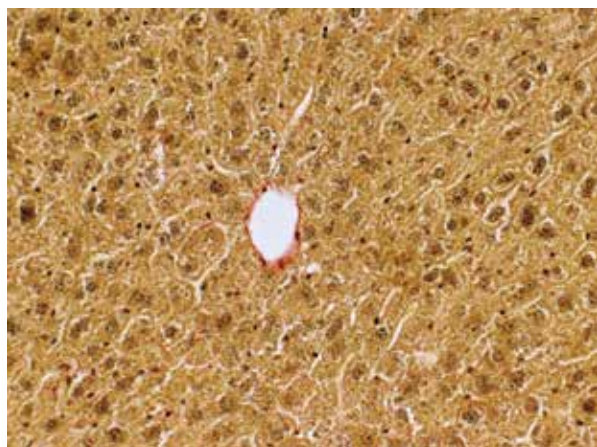

AHF

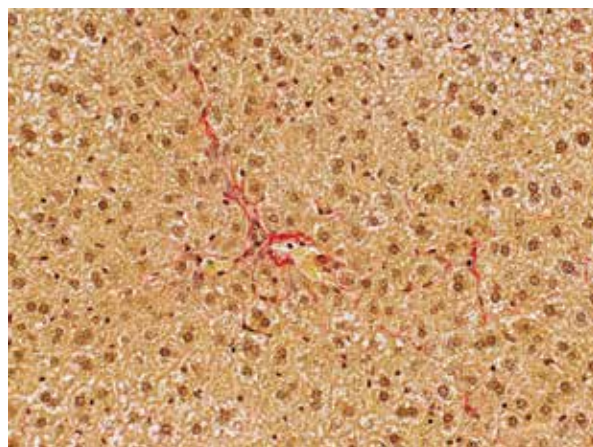

AHF+EPA

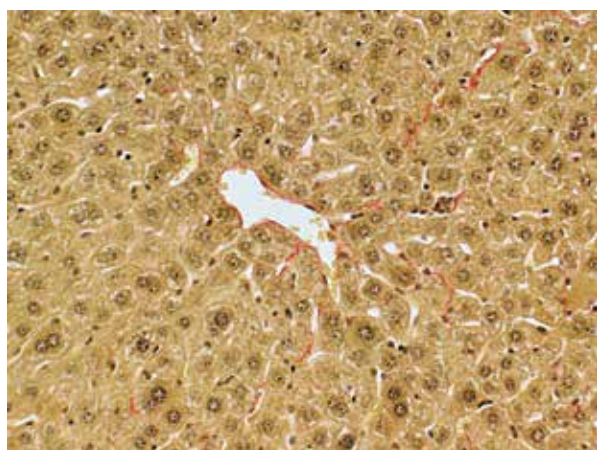

AHF+DHA

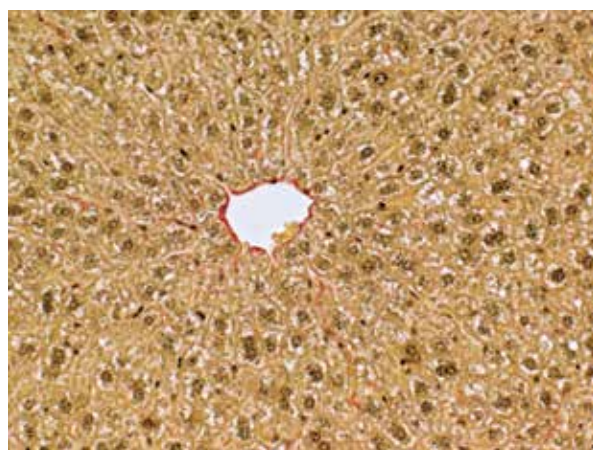

Supplement: S1 Fig — (PDF) [file pone.0157580.s001.pdf]
