# Supplementary material for: Different Effects of Eicosapentaenoic and Docosahexaenoic Acids on Atherogenic High-Fat Diet-Induced Non-Alcoholic Fatty Liver Disease in Mice
Source: PLoS One. 2016 Jun 22;11(6):e0157580. doi: 10.1371/journal.pone.0157580 (PMC4917109; doi:10.1371/journal.pone.0157580)
Supplement: S1 Table — (PDF) [file pone.0157580.s002.pdf]

Table S1

The Composition of the Atherogenic High-Fat (AHF) Diet

Composition	%
Casein	25.000
L-cystin	0.375
Corn starch	13.119
Sucrose	20.000
Soybean oil	2.000
Cellurose	5.000
Lard	28.000
Cholesterol	1.250
Cholic acid	0.500
Mineral mix AIN93G	3.500
Vitamin mix AIN93	1.000
Choline bitartrate	0.250
tert-Butylhydroquinone	0.006
Total	100.000
