# Supplementary material for: Different Effects of Eicosapentaenoic and Docosahexaenoic Acids on Atherogenic High-Fat Diet-Induced Non-Alcoholic Fatty Liver Disease in Mice
Source: PLoS One. 2016 Jun 22;11(6):e0157580. doi: 10.1371/journal.pone.0157580 (PMC4917109; doi:10.1371/journal.pone.0157580)
Supplement: S2 Table — (PDF) [file pone.0157580.s003.pdf]

Table S2

Fatty acid composition (mol% of total fatty acids) of the chow and AHF diet.

	Chow	AHF
C12:0	0.1	0.2
C14:0	0.6	2.0
C16:0	14.6	24.8
C16:1n-7	0.9	2.8
C18:0	2.5	14.8
C18:1n-9	23.1	44.0
C18:2n-6	46.8	9.8
C18:3n-6	ND	ND
C18:3n-3	3.8	0.8
C20:0	0.4	0.2
C20:1n-9	1.6	0.5
C20:2n-6	ND	0.3
C20:3n-9	ND	ND
C20:3n-6	ND	0.1
C20:4n-6	0.1	0.1
C20:5n-3	1.8	ND
C22:0	0.4	0.05
C22:1n-9	0.2	ND
C22:4n-6	ND	ND
C22:5n-3	0.2	ND
C24:0	0.5	0.01
C22:6n-3	2.3	ND
C24:1n-9	0.1	ND

ND; not detected.
